# Patients' prognosis of intrahepatic cholangiocarcinoma and combined hepatocellular‐cholangiocarcinoma after resection

**DOI:** 10.1002/cam4.2495

**Published:** 2019-08-13

**Authors:** Peipei Song, Yutaka Midorikawa, Hisashi Nakayama, Tokio Higaki, Masamichi Moriguchi, Osamu Aramaki, Shintaro Yamazaki, Masaru Aoki, Kenichi Teramoto, Tadatoshi Takayama

**Affiliations:** ^1^ Department of Digestive Surgery Nihon University School of Medicine Tokyo Japan

**Keywords:** cHCC‐CC, ICC, prognosis, tumor number, tumor size, vascular invasion

## Abstract

Combined hepatocellular‐cholangiocarcinoma (cHCC‐CC) and intrahepatic cholangiocarcinoma (ICC) are classified into one category, but comparison of prognosis of the two carcinomas remains controversial. The aim of the current study was to investigate surgical outcomes for patients with ICC or cHCC‐CC who underwent resection in order to elucidate whether the classification of ICC and cHCC‐CC is justified. Subjects were 61 patients with ICC and 29 patients with cHCC‐CC who underwent liver resection from 2001 to 2017. Clinic‐pathological data from the two groups were compared. Tumor number and vascular invasion were independent risk factors for recurrence‐free survival (RFS) in both groups (*P* < .001 for both). Of note, for patients with ICC, tumor cut‐off size of 5 cm showed statistical significance in median RFS (>5 cm vs ≤5 cm, 0.5 years vs 4.0 years, *P* = .003). For patients with cHCC‐CC, tumor cut‐off size of 2 cm showed statistical significance in median RFS (>2 cm vs ≤2 cm, 0.6 years vs 2.6 years, *P* = .038). The median RFS of patients with cHCC‐CC was 0.9 years (95% confidence interval: 0.3‐1.6), which was poorer than that of patients with ICC (1.3 years, 0.5‐2.1) (*P* = .028); the rate of RFS at 5 years was 0% and 37.7% respectively. Our study supports the concept of classifying ICC and cHCC‐CC into different categories because of a significant difference in RFS between the two.

## Graphical abstract

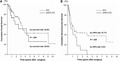

## 1 INTRODUCTION

Intrahepatic cholangiocarcinoma (ICC) is the second most common primary liver tumor following hepatocellular carcinoma (HCC) and accounts for 5‐15% of cases of primary liver cancer.^1^ Combined hepatocellular‐cholangiocarcinoma (cHCC‐CC) originates from cells that have the histological features of HCC and CC, with an incidence rate from 0.4% to 14.2% in different regions.^2^, ^3^, ^4^, ^5^, ^6^

Patients with cHCC‐CC are most often diagnosed based on pathological findings, and after surgery in particular. The 7th and 8th editions of the American Joint Committee on Cancer (AJCC) staging^7^, ^8^ and International Union for Cancer Control (UICC) tumor‐nodes‐metastasis staging^9^, ^10^ classify cHCC‐CC and ICC into one category, but there is debate over the clinical features of cHCC‐CC in comparison to those of ICC and assessment of their prognosis. Some studies have suggested that patients with cHCC‐CC have a poorer prognosis than those with ICC,^3^, ^11^ while others have either reported the opposite^12^ or no significant difference in survival.^2^, ^13^, ^14^ Liver transplantation is also one of choice for ICC and cHCC‐CC.^15^, ^16^, ^17^, ^18^ However, surgical outcomes of ICC patients after liver transplantation is quite poor due to higher recurrence and shorter survival compared to HCC patients.^17^ Liver transplantation also cannot contribute to better prognosis of patients with cHCC‐CC compared to those with HCC, but may improve surgical outcomes than ICC patients.^15^

Furthermore, the previous edition of the AJCC/UICC staging system excluded tumor size as a prognostic factor from the tumor (T) classification,^7^, ^9^ but the current (8th) edition clearly indicates that a tumor size of 5 cm is a factor for determining the T classification of ICC.^8^, ^10^ Besides, based on the HCC staging system,^19^ the Liver Cancer Study Group of Japan (LCSGJ) staging system determines the T classification based on a tumor size of 2 cm, the tumor number, and the presence of vascular/serosal invasion. A nationwide study by the LCSGJ revealed that a solitary ICC ≤2 cm in size without vascular or major biliary invasion can have an excellent prognosis.^20^

In order to elucidate whether classifying ICC and cHCC‐CC into one category is justified, the current study investigated the clinical features of and prognosis for the two types of liver cancer.

## 2 PATIENTS AND METHODS

### 2.1 Study population

**Figure 1:**
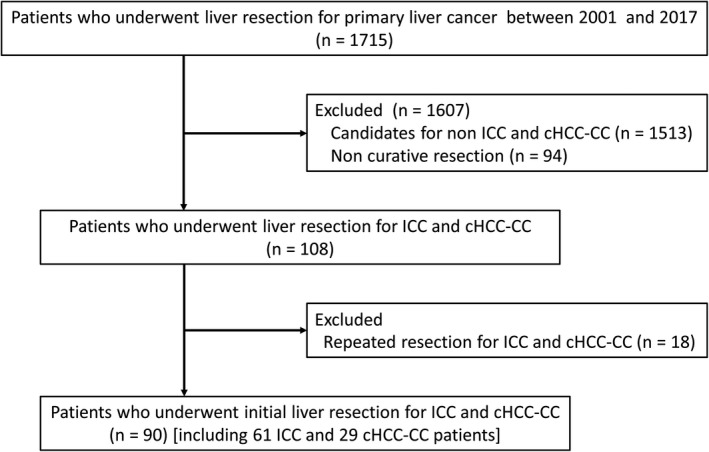
Flow‐chart for patient selection. cHCC‐CC, combined hepatocellular‐cholangiocarcinoma; ICC, intrahepatic cholangiocarcinoma

Data from patients with primary liver cancer undergoing liver resection at the Department of Digestive Surgery of Nihon University Itabashi Hospital in Tokyo from 2001 to 2017 were retrospectively analyzed. Of them, the patients with ICC or cHCC‐CC who underwent initial and curative resection were included in this study (Figure 1).

For consecutive patients with ICC or cHCC‐CC, the diagnosis was based on imaging studies and clinical data, and confirmed by pathological findings. Combined hepatocellular‐cholangiocarcinoma was classified as the mixed type in accordance with the Allen and Lisa classification.^21^ Clinical and pathological data on patients were retrospectively collected. This study was approved by this facility's research ethics committee.

### 2.2 Preoperative evaluation

A routine preoperative evaluation for primary liver cancer was performed.^20^, ^22^, ^23^, ^24^ Computed tomography (CT) and gadolinium‐ethoxybenzyl‐diethylenetriamine pentaacetic acid‐enhanced magnetic resonance imaging (EOB‐MRI) were performed to evaluate the characteristics of each condition such as tumor size, tumor number, the presence of vascular invasion, and the presence of extrahepatic metastasis. Tumor markers including alpha‐fetoprotein (AFP), lens culinaris agglutinin‐reactive AFP (AFP‐L3), des‐gamma‐carboxy prothrombin (DCP), carcinoembryonic antigen (CEA), and carbohydrate antigen 19‐9 (CA 19‐9) were evaluated.

### 2.3 Surgical procedures

All patients underwent open liver resection, and hilar lymphadenectomy was not routinely performed unless lymph node metastasis was suspected preoperatively. The indications for liver resection and the procedure were determined by assessing the liver functional reserve according to Makuuchi's criteria for liver resection.^25^ Briefly, liver resection is contraindicated in patients who have refractory ascites, hyperbilirubinemia (>2 mg/dL), or both. The extent of liver resection is determined on the basis of the serum total bilirubin level and indocyanine green retention rate at 15 minutes (ICG‐R_15_) value. Anatomical resection of Couinaud's segment was the first‐line operative procedure. Major liver resection was defined as removal of two or more segments, and minor resection was defined as resection of not more than one segment. R0 resection was defined as liver resection with “clear resection margin”, and R1 as liver resection with “resection margin touching inked tumor”.^26^ Patients undergoing R2 resection were excluded in this study.

### 2.4 Postoperative pathological factors

Each pathological factor was defined in accordance with the *General Rules for the Clinical and Pathological Study of Primary Liver Cancer*.^19^ Tumor size and number were determined based on pathological findings. Based on the LCSGJ classification system,^19^ the impact of portal vein invasion, hepatic vein invasion, arterial invasion, biliary invasion, and serosal invasion was evaluated according to the microscopic grade of each factor. Lymph node metastasis, distant metastasis, and intrahepatic metastasis were also analyzed.

### 2.5 Postoperative follow‐up

In general, patients were followed up every 1‐3 months during the first year after surgery and every 3 months thereafter. Levels of tumor markers such as AFP, DCP, CEA, and CA19‐9 were measured, and imaging studies, including CT and ultrasonography, were performed every 3 months on all patients. Recurrence was identified based on dynamic CT and/or EOB‐MRI. In patients with recurrent ICC or cHCC‐CC, the time between the date of surgery and recurrence was defined as the recurrence‐free period. Recurrent ICC or cHCC‐CC was treated with repeated liver resection, transcatheter arterial chemoembolization (TACE), radiofrequency ablation, or chemotherapy depending on the status of the ICC or cHCC‐CC and liver function at the time of recurrence. The final follow‐up was completed on September 12, 2018.

### 2.6 Statistical analysis

Continuous variables are expressed as the median and range and were compared using the Mann‐Whitney *U* test. Categorical variables are expressed as frequencies and percentages and were compared using the Chi‐square test. Cumulative survival curves were plotted using the Kaplan‐Meier method, and curves were compared using the log‐rank test. The predictive ability of tumor size was assessed by receiver operating characteristic (ROC) curve analysis and the corresponding area under the curve (AUC). The optimal cut‐off size was set as the value maximizing the sum of sensitivity and specificity.

The significance of clinical and pathological characteristics was assessed using univariate analysis. Variables that were significantly associated with survival and recurrence‐free survival (RFS) in univariate analysis were subjected to multivariate analysis using Cox's proportional hazards regression model (with a backward stepwise procedure), and the corresponding 95% confidence intervals (CI) were calculated. Two‐tailed *P* values <.05 were considered statistically significant.

Statistical analysis was performed using SPSS version 22.0 (SPSS Inc).

## 3 RESULTS

### 3.1 Clinicopathological features

Based on pathological findings, 61 patients were diagnosed with ICC and 29 patients were diagnosed with cHCC‐CC (Figure 1). Levels of both AFP (*P* < .001) and AFP‐L3 (*P* = .006) were higher in patients with cHCC‐CC compared to levels in patients with ICC. The number of R1 was more frequent in cHCC‐CC group, but there was no significance between the two groups (cHCC‐CC vs ICC, 27.6% vs 24.6%, *P* = .799). The median tumor size was 4.0 cm (range: 1.0‐25.0 cm) for ICC and 3.0 cm (range: 1.0‐11.0 cm) for cHCC‐CC (Table 1).

**Table 1:** Baseline characteristics of patients with ICC or cHCC‐CC

Variables, n (%)	Total patients (n = 90)	ICC (n = 61)	cHCC‐CC (n = 29)	*P* values
Age (y)^a^	68 (23‐84)	69 (23‐84)	64 (45‐80)	.080
Gender, male	64 (71.1)	41 (67.2)	23 (79.3)	.321
Underlying liver disease				<.001
Normal liver	36 (40.0)	31 (50.8)	5 (17.2)	
Chronic hepatitis	41 (45.6)	27 (44.3)	14 (48.3)	
Liver cirrhosis	13 (14.4)	3 (4.9)	10 (34.5)	
Tumor markers^a^				
AFP (ng/mL)	4.2 (0.8‐3689.4)	3.7 (0.8‐808.1)	10.9 (1.3‐3689.4)	<.001
AFP‐L3 (%)	0.5 (0.0‐90.9)	0.5 (0‐90.6)	5.7 (0.0‐90.9)	.006
DCP (mAU/mL)	21.0 (1.0‐15933.0)	20.0 (1.0‐13380.0)	26.0 (9.0‐15933.0)	.089
CEA (ng/mL)	3.0 (0.2‐175.1)	3.6 (1.1‐175.1)	2.6 (0.2‐32.7)	.067
CA 19‐9 (U/mL)	31.3 (0.1‐117800.0)	31.3 (0.1‐117800.0)	35.2 (0.1‐915.0)	.626
ICR‐R15 (%)^a^	10.0 (1.9‐34.0)	9.9 (1.9‐33.4)	11.5 (5.16‐34.0)	.113
Child‐Pugh Score, A	83 (92.2)	55 (90.2)	28 (96.6)	.421
Operating time (min)^a^	384.5 (145.0‐869.0)	419.0 (191.0‐869.0)	315.0 (145.0‐567.0)	.024
Amount of bleeding (mL)^a^	366.0 (29.0‐11002.0)	364.0 (29.0‐11002.0)	385.0 (35.0‐2740.0)	1.000
Blood transfusion, performed	9 (10.0)	7 (11.5)	2 (6.9)	.712
Extent of resection, major	46 (51.1)	39 (63.9)	7 (24.1)	.001
Surgical margin, R1	23 (25.6)	15 (24.6)	8 (27.6)	.799
Tumor number, solitary	72 (80.0)	49 (80.3)	23 (79.3)	1.000
Tumor size^a^	3.5 (1.0‐25.0)	4.0 (1.0‐25.0)	3.0 (1.0‐11.0)	.070
Vascular invasion, present	41 (45.6)	28 (45.9)	13 (44.8)	1.000
Serosal invasion, present	22 (24.4)	19 (31.1)	3 (10.7)	.037
Lymph node metastases, present	10 (11.1)	6 (9.8)	4 (13.8)	.721
Distant metastasis, present	1 (1.1)	0 (0.0)	1 (3.4)	.322
Intrahepatic metastasis, present	13 (14.4)	9 (14.8)	4 (13.8)	1.000
Histologic differentiation, poor	31 (34.4)	12 (19.7)	19 (65.5)	<.001
Recurrence, present	50 (55.6)	29 (47.5)	21 (72.4)	.040

Abbreviations: AFP, alpha‐fetoprotein; AFP‐L3, lens culinaris agglutinin‐reactive alpha‐fetoprotein; CA 19‐9, carbohydrate antigen 19‐9; CEA, carcinoembryonic antigen; cHCC‐CC, combined hepatocellular‐cholangiocarcinoma; DCP, des‐gamma‐carboxy prothrombin; ICC, intrahepatic cholangiocarcinoma; ICR‐R15, indocyanine green retention rate at 15 min.

a Median (range).

### 3.2 Patient survival

After a median follow‐up of 1.2 years (range: 0.1‐10.5 years) for 90 patients, recurrence was noted in 50 patients (55.6%), including 29 (47.5%) with ICC and 21 (72.4%) with cHCC‐CC (*P* = .040).

**Figure 2:**
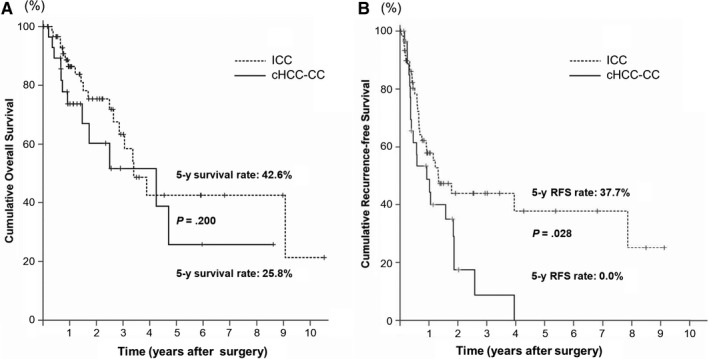
The cumulative OS (A) and RFS (B) curves for patients with ICC and patients with cHCC‐CC who underwent resection. cHCC‐CC, combined hepatocellular‐cholangiocarcinoma; ICC, intrahepatic cholangiocarcinoma; OS, overall survival; RFS, recurrence‐free survival

The median overall survival (OS) of 61 patients with ICC was 3.4 years (95% CI: 2.4‐4.4) and that of 29 patients with cHCC‐CC was 4.2 years (95% CI: 0.9‐7.6) (*P* = .200). The median RFS was 1.3 years (95% CI: 0.5‐2.1) for patients with ICC and 0.9 years (95% CI: 0.3‐1.6) for patients with cHCC‐CC (*P* = .028). The rates of OS and RFS at 5 years were 42.6% and 37.7%, respectively, in patients with ICC; and 25.8% and 0%, respectively, in patients with cHCC‐CC (Figure 2).

### 3.3 Prognostic factors

Cox's proportional model indicated that tumor number was the strongest independent risk factor for OS and RFS in both groups. Vascular invasion was also an independent risk factor for RFS in both groups (Tables 2 and 3).

**Table 2:** Univariate and multivariate analysis of prognostic factors in patients with ICC

Variables	Cases	Univariate analysis		Multivariate analysis		Univariate analysis		Multivariate analysis	
		OS n (%)	*P*	HR (95% CI)	*P*	RFS n (%)	*P*	HR (95% CI)	*P*
Age >65 y	39	28 (71.8)	.783			24 (61.5)	.056		
Gender, male	41	31 (75.6)	.127			21 (51.2)	.619		
Underlying liver disease, present^a^	30	18 (60.0)	.201			14 (46.7)	.352		
AFP >20 ng/mL	6	3 (50.0)	.185			2 (33.3)	.234		
AFP‐L3 >10%	8	4 (50.0)	.082			5 (62.5)	.713		
DCP >40 mAU/mL	5	1 (20.0)	.074			2 (40.0)	.963		
CEA >5 ng/mL	20	14 (70.0)	.770			10 (50.0)	.778		
CA 19‐9 >40 U/mL	27	17 (63.0)	.094			13 (48.1)	.183		
ICR‐R15 >10%	30	22 (73.3)	.132			18 (60.0)	.047		.261
Child‐Pugh Score, A	55	37 (67.3)	.533			27 (49.1)	.109		
Operating time >419 min^b^	30	20 (66.7)	.439			13 (43.3)	.695		
Amount of bleeding >364 mL^b^	30	22 (73.3)	.556			15 (50.0)	.949		
Blood transfusion, Performed	7	2 (28.6)	.026		.522	3 (42.9)	.796		
Extent of resection, major	39	28 (71.8)	.803			22 (56.4)	.483		
Surgical margin, R1	15	11 (73.3)	.605			7 (46.7)	.942		
Tumor number, solitary	49	39 (79.6)	<.001	22.929 (6.018‐87.365)	<.001	31 (63.3)	<.001	15.013 (5.321‐42.359)	<.001
Tumor size ≤ 2 cm	8	7 (87.5)	.364			6 (75.0)	.274		
Tumor size ≤ 5 cm	39	32 (82.1)	.003		.481	25 (64.1)	.003		.673
Vascular invasion, present	28	16 (57.1)	.001		.122	11 (39.3)	.002	2.574 (1.136‐5.832)	.023
Serosal invasion, present	19	10 (52.6)	.159			7 (36.8)	.285		
Lymph node metastases, present	6	3 (50.0)	.126			2 (33.3)	.169		
Intrahepatic metastasis, present	9	2 (22.2)	<.001		.606	1 (11.1)	<.001		.413
Histologic differentiation, poor	12	10 (83.3)	.922			7 (58.3)	.657		

Abbreviations: CI, confidence interval; HR, hazard ratio; ICC, intrahepatic cholangiocarcinoma; ICG‐R15, indocyanine green retention rate at 15 min; OS, overall survival; RFS, recurrence‐free survival.

a Chronic hepatitis or liver cirrhosis.

b Median value.

**Table 3:** Univariate and multivariate analysis of prognostic factors in patients with cHCC‐CC

Variables	Cases	Univariate analysis		Multivariate analysis		Univariate analysis		Multivariate analysis	
		OS n (%)	*P*	HR (95% CI)	*P*	RFS n (%)	*P*	HR (95% CI)	*P*
Age >65 y	14	6 (42.9)	.295			3 (21.4)	.440		
Gender, male	23	13 (56.5)	.525			7 (30.4)	.796		
Underlying liver disease, present^a^	24	14 (58.3)	.843			7 (29.2)	.184		
AFP >20 ng/mL	11	6 (54.5)	.047		.335	3 (27.3)	.005		.696
AFP‐L3 >10%	12	8 (66.7)	.260			4 (33.3)	.019		.785
DCP >40 mAU/mL	11	7 (63.6)	.768			4 (36.4)	.855		
CEA >5 ng/mL	5	0 (0.0)	.001		.078	0 (0.0)	.006		.065
CA 19‐9 >40 U/mL	13	6 (46.2)	.182			3 (23.1)	.325		
ICR‐R15 >10%	15	7 (46.7)	.325			2 (13.3)	.728		
Child‐Pugh Score, A	28	17 (60.7)	.629			8 (28.6)	.865		
Operating time >315 min^b^	14	8 (57.1)	.965			4 (28.6)	.590		
Amount of bleeding >385 mL^b^	14	8 (57.1)	.964			4 (28.6)	.506		
Blood transfusion, performed	2	1 (50.0)	.646			1 (50.0)	.932		
Extent of resection, minor	22	13 (59.1)	.231			7 (31.8)	.015	4.063 (1.223‐13.497)	.022
Surgical margin, R1	8	5 (62.5)	.516			2 (25.0)	.656		
Tumor number, solitary	23	15 (65.2)	.002	7.382 (1.628‐33.468)	.010	7 (30.4)	<.001	10.631 (2.553‐44.273)	.001
Tumor size >2 cm^c^	24	13 (54.2)	.259			4 (16.7)	.038		.188
Vascular invasion, present	13	6 (46.2)	.228			3 (23.1)	.036	3.247 (1.023‐10.299)	.046
Serosal invasion, present	3	3 (100.0)	.310			0 (0.0)	.325		
Lymph node metastases, present	4	3 (75.0)	.694			1 (25.0)	.819		
Intrahepatic metastasis, present	4	1 (25.0)	.083			0 (0.0)	.200		
Histologic differentiation, poor	19	9 (47.4)	.378			6 (31.6)	.411		

Abbreviations: AFP, alpha‐fetoprotein; AFP‐L3, lens culinaris agglutinin‐reactive alpha‐fetoprotein; cHCC‐CC, combined hepatocellular‐cholangiocarcinoma; CI, confidence interval; HR, hazard ratio; OS, overall survival; RFS, recurrence‐free survival.

a Chronic hepatitis or liver cirrhosis.

b Median value.

c A tumor cut‐off size of 5 cm (*P* = .001) was also significantly associated with RFS according to univariate analysis; when a tumor cut‐off size of 5 cm was used in multivariate analysis, multiple tumors (HR, 11.463; 95% CI, 2.536‐51.819; *P* = .002) and a tumor size > 5 cm (HR, 6.240; 95% CI, 1.619‐24.054; *P* = .008) were identified as significant predictors of RFS.

**Figure 3:**
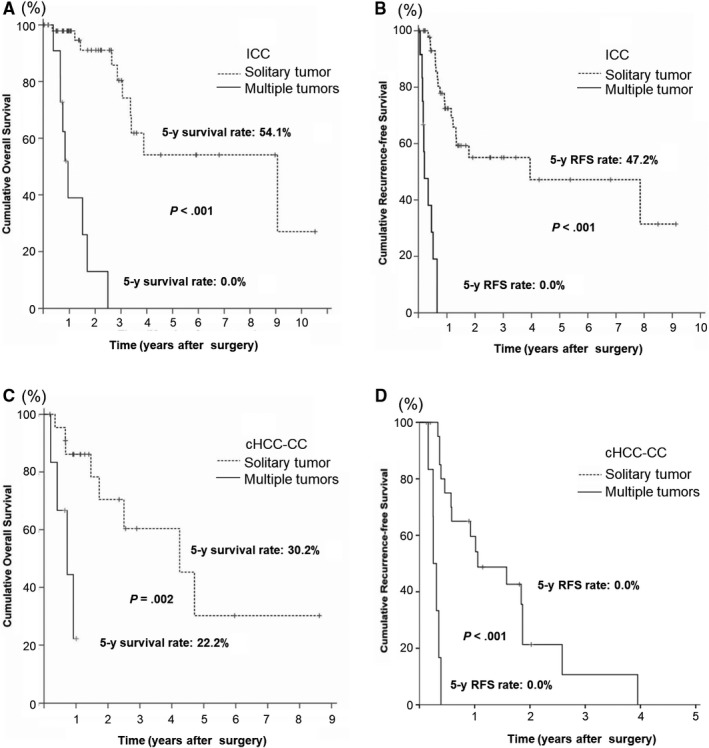
The cumulative OS and RFS curves for patients with ICC (A, B) and patients with cHCC‐CC (C, D) stratified by tumor number. cHCC‐CC, combined hepatocellular‐cholangiocarcinoma; ICC, intrahepatic cholangiocarcinoma; OS, overall survival; RFS, recurrence‐free survival

In ICC patients with multiple tumors, the rates of OS and RFS at 5 years were 0% respectively, which were lower than those of patients with a solitary tumor (54.1% and 47.2%, respectively; *P* < .001 for both) (Figure 3A,B). In ICC patients with vascular invasion, the rate of RFS at 5 years was 20.4%, which was lower than those of patients without vascular invasion (52.7%) (*P* = .002).

In cHCC‐CC patients with multiple tumors, the rate of OS at 5 years was 22.2%, which was lower than those of patients with a solitary tumor (30.2%, *P* = .002) (Figure 3C). The RFS at 5 years were poor in both groups. The median RFS was 0.3 years (95% CI: 0.2‐0.4) in patients with multiple tumors, which was shorter than 1.1 years (95% CI: 0.2‐1.9) in patients with a solitary tumor (*P* < .001) (Figure 3D). The median RFS was 0.4 years (95% CI: 0.1‐0.7) in patients with vascular invasion, which was shorter than 1.8 years (95% CI: 1.1‐2.6) in patients without vascular invasion (*P* = .019).

The resection margin status was not identified as an independent risk factor for OS and RFS in ICC and cHCC‐CC (*P* > .05, respectively) (Tables 2 and 3). The extent of resection was identified as an independent risk factor for RFS in patients with cHCC‐CC (*P* = .022) (Table 3). In cHCC‐CC patients undergoing major resection, the rate of RFS at 5 years was 0%, which was lower than those of patients undergoing a minor resection (10.8%, *P* = .022).

### 3.4 Tumor size and prognosis

When patients were stratified using a tumor cut‐off size of 2 cm, the prognosis for patients with ICC did not differ significantly. However, a tumor cut‐off size of 5 cm was significantly associated with OS and RFS (*P* = .003 for both) according to a univariate analysis of patients with ICC (Table 2). In patients with an ICC tumor >5 cm in size, the median OS and RFS were 2.5 years (95% CI: 0.7‐4.2) and 0.5 years (95% CI: 0.2‐0.7), respectively; the median OS and RFS were shorter than those in patients with a tumor ≤5 cm in size (6.5 years [95% CI: 4.8‐8.3] and 4.0 years [95% CI: 0.1‐8.1], respectively) (*P* = .003 for both). The OS and RFS rates at 5 years were 21.7% and 28.1% in patients with an ICC tumor >5 cm in size and 59.5% (*P* = .001) and 44.3% (*P* = .002) in patients with a tumor ≤5 cm in size.

In contrast, a tumor cut‐off size of 2 cm was significantly associated with RFS (*P* = .038) according to a univariate analysis of patients with cHCC‐CC (Table 3). In patients with a cHCC‐CC tumor >2 cm in size, the median RFS was 0.6 years; the median RFS was shorter than that of patients with a tumor ≤2 cm in size (2.6 years, *P* = .038).

In order to determine the optimal cut‐off value of tumor size for survivals, we performed ROC curve analysis (Figure S1). The area under the ROC curve was 0.706 (95% CI: 0.568‐0.844; *P* = .010) for OS of ICC patients. The optimal cut‐off value of tumor size was 4.55 cm, with sensitivity of 63.2% and specificity of 81.0%. On the other hand, the optimal cut‐off size was 2.35 cm for OS of cHCC‐CC patients, with sensitivity of 91.7% and specificity of 41.2% (Table 4).

**Table 4:** The clinical utility of tumor size with different cut‐off values in predicting survival and recurrence for patients with ICC and cHCC‐CC

Tumor cut‐off values	Sensitivity (%)	Specificity (%)	Youden index (%)	Area under ROC curve (95% CI)	*P*
ICC survival					
Optimal cut‐off size: 4.55 cm	63.2	81.0	44.1	0.706 (0.568‐0.844)	.010
Cut‐off size of 2 cm	94.7	16.7	11.4		
Cut‐off size of 5 cm	63.2	76.2	39.3		
ICC recurrence					
Optimal cut‐off size: 3.75 cm	72.4	62.5	34.9	0.683 (0.548‐0.818)	.014
Cut‐off size of 2 cm	93.1	18.7	11.9		
Cut‐off size of 5 cm	51.7	78.1	29.8		
cHCC‐CC survival					
Optimal cut‐off size:2.35 cm	91.7	41.2	32.8	0.588 (0.376‐0.801)	.425
Cut‐off size of 2 cm	91.7	29.4	21.1		
Cut‐off size of 5 cm	25.0	82.4	7.4		
cHCC‐CC recurrence					
Optimal cut‐off size: 2.15 cm	95.2	62.5	57.7	0.708 (0.442‐0.974)	.088
Cut‐off size of 2 cm	95.2	62.5	57.7		
Cut‐off size of 5 cm	23.8	87.5	11.3		

Abbreviations: cHCC‐CC, combined hepatocellular‐cholangiocarcinoma; ICC, intrahepatic cholangiocarcinoma.

## 4 DISCUSSION

Even though the 7th and 8th editions of the AJCC/UICC staging system classify cHCC‐CC and ICC into one category,^7^, ^8^, ^9^, ^10^ patients with cHCC‐CC had a poorer prognosis than those with ICC and the prognoses for the two types of liver cancer differ when stratified by the tumor size in the current study.

Differences in prognosis for ICC and cHCC‐CC may be due to their distinct mechanisms of carcinogenesis and biological behavior. The disadvantageous behavior of cHCC‐CC may be related to tumor cells that originate from pluripotent hepatic precursor cells.^27^, ^28^ According to one hypothesis, the ICC components in cHCC‐CC do not originate from standard HCC and hepatic progenitor cells that undergo malignant transformation; instead, those cells may exhibit dual differentiation.^29^, ^30^, ^31^, ^32^ In the current study, chronic hepatitis or liver cirrhosis and poorly differentiated cancer were more frequently noted in patients with cHCC‐CC. Consequently, the rate of recurrence was higher in the cHCC‐CC group. Differences in prognosis for ICC and cHCC‐CC may be due to differences in the aforementioned background liver factors and the grade of cancer differentiation. Therefore, it might be justified to propose that ICC and cHCC‐CC be clinically regarded as different categories.

Tumor number is regarded as one of the important factors for determining the T classification according to the current staging systems of the AJCC/UICC^8^, ^10^ and LCSGJ.^19^ A greater number of tumors may result in satellite nodules or intrahepatic metastasis, both of which are reported to be associated with a worse outcome.^33^, ^34^, ^35^, ^36^ Consistent to the previous study, our data showed that tumor number was a significant predictor of OS and RFS in both groups, and patients with multiple ICCs had a poorer prognosis than those with a solitary tumor. It was also true for patients with cHCC‐CC. Similarly, numerous studies have reported on the prognostic importance of vascular invasion in ICC and cHCC‐CC.^6^, ^14^, ^37^, ^38^ In the current study, vascular invasion was also identified as a significant predictor of RFS in both groups. Patients with ICC with vascular invasion had a poorer prognosis than those without vascular invasion, and it was also true for patients with cHCC‐CC.

A point worth noting is that the prognostic importance of tumor size in patients with ICC or cHCC‐CC is still a subject of debate. Based on the analysis of population‐based data of patients with ICC in the United States, a study indicated that tumor size was not a prognostic factor, and it was unable to confirm the prognostic impact of a tumor cut‐off size of 2 or 5 cm on survival.^39^ Using Japanese data from the LCSGJ, a report indicated that an ICC cut‐off size of 2 cm in the largest dimension readily predicted patient survival and that a solitary ICC ≤2 cm in size without vascular or major biliary invasion can have an excellent prognosis.^20^ Several studies have similarly revealed the prognostic importance of tumor size in patients with cHCC‐CC,^6^, ^40^, ^41^ while others have found that tumor size was not associated with survival.^42^, ^43^ The reason why the prognostic impact of a tumor cut‐off size of 2 or 5 cm differs in the literature is unclear, but detecting a tumor ≤2 cm in size is very difficult due to the lack of symptoms. Surveillance is crucial for patients with hepatic disease. Moreover, the prognostic role of tumor size needs to be evaluated in additional studies.

In the current study, tumor size was significantly associated with prognosis in both groups according to univariate analysis. However, the prognoses of ICC and cHCC‐CC differed when the two forms of liver cancer were stratified according to tumor size. When stratified by a tumor cut‐off size of 2 cm, the prognosis for patients with ICC did not differ significantly. However, patients with cHCC‐CC >2 cm in size had a poorer prognosis than those with cHCC‐CC ≤2 cm in size. When stratified by a tumor cut‐off size of 5 cm, patients with ICC >5 cm in size had a poorer prognosis than those with ICC ≤5 cm in size. Given that the optimal cut‐off values of tumors size were approximately 5 cm for patients with ICC and 2 cm for those with cHCC‐CC in this study, tumor factor defined by AJCC/UICC and LCSGJ staging systems maybe appropriate for ICC and cHCC‐CC, respectively. However, patient number was too small in this study, and therefore it should be determined using a large cohort such as nationwide study.

The current study had several limitations. First, the current study was a retrospective, single‐center study. Consequently there were some differences of the background between the ICC and cHCC‐CC patients. Although propensity‐score matching is undoubtedly one of the best solution methods for observational data, the sample size was not large enough due to the low prevalence of ICC and cHCC‐CC, and therefore the statistical matching was impossible. Second, all patients in this study underwent surgical resection. Given that substantial proportion of patients with ICC and cHCC‐CC are not to be candidates for resection, the clinical features and prognosis for patients receiving nonsurgical treatment should be evaluated and compared.

## 5 CONCLUSION

The current study revealed that patients with cHCC‐CC had a poorer prognosis than those with ICC and that the prognosis differed significantly when cHCC‐CC was stratified by a tumor cut‐off size of 2 cm and when ICC was stratified by a tumor cut‐off size of 5 cm. This difference is likely to be due to the difference in the biological behavior of the two types of carcinoma. Taken together, the current findings suggest that ICC and cHCC‐CC should be classified into different categories. Moreover, the differences in tumor size, tumor characteristics, and tumor biology need to be further evaluated in order to accurately predict the prognosis for patients with ICC or cHCC‐CC.

## CONFLICT OF INTEREST

There is no conflict of interest to disclose.
