# Supplementary figures and images for: Patients' prognosis of intrahepatic cholangiocarcinoma and combined hepatocellular‐cholangiocarcinoma after resection
Source: Cancer Med. 2019 Aug 13;8(13):5862–71. doi: 10.1002/cam4.2495 (PMC6792494; doi:10.1002/cam4.2495)

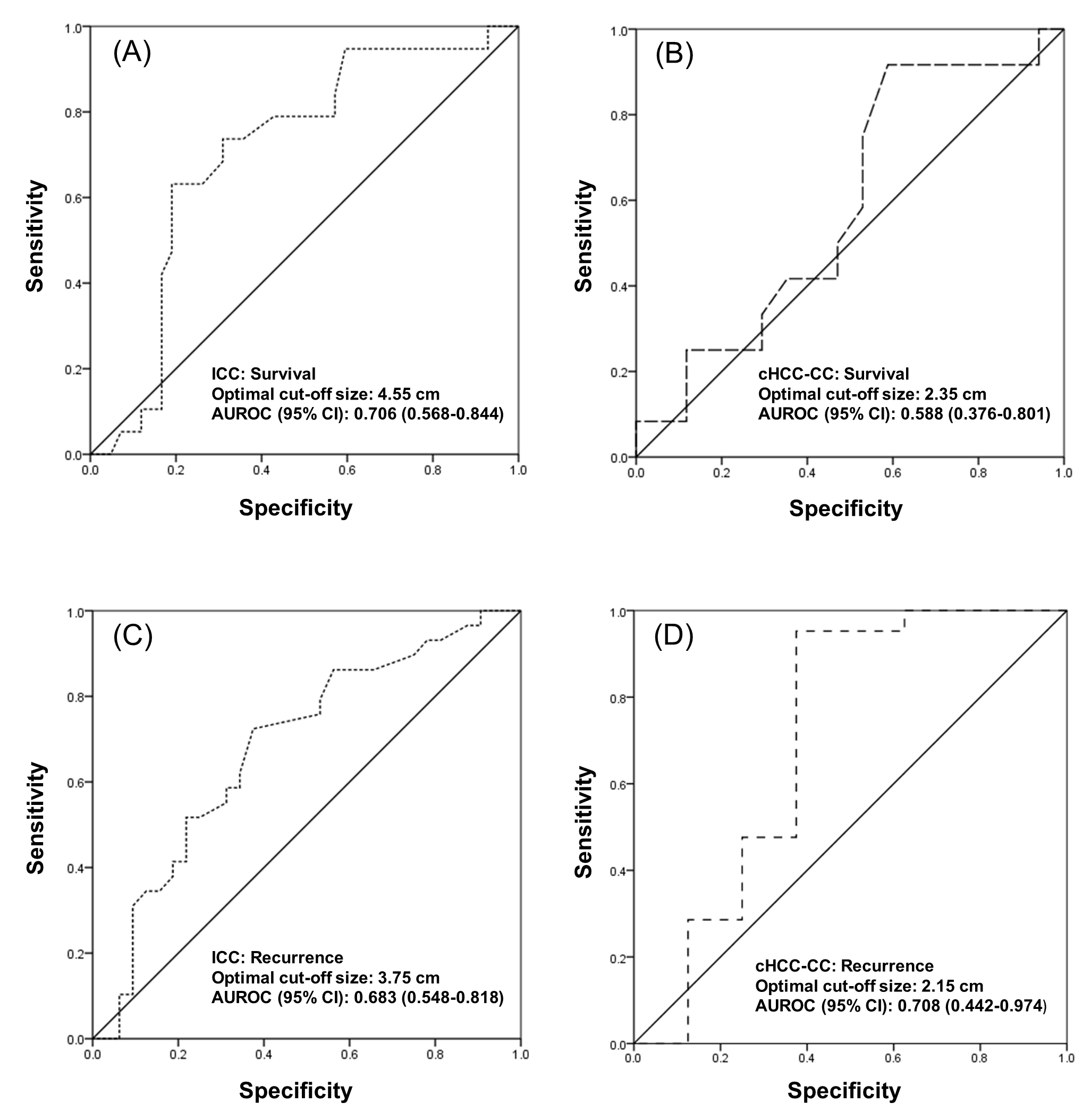

Supplement: Supplementary file 1 [file CAM4-8-5862-s001.tif]
