# Supplementary material for: Patients' prognosis of intrahepatic cholangiocarcinoma and combined hepatocellular‐cholangiocarcinoma after resection
Source: Cancer Med. 2019 Aug 13;8(13):5862–71. doi: 10.1002/cam4.2495 (PMC6792494; doi:10.1002/cam4.2495)
Supplement: Supplementary file 2 [file CAM4-8-5862-s002.docx]

**Figure S1.** ROC curve comparing tumor cut-off size predicting survival (A and B) and recurrence (C and D) in patients with ICC and cHCC-CC. ICC, intrahepatic cholangiocarcinoma; cHCC-CC, combined hepatocellular-cholangiocarcinoma; ROC, receiver operating characteristic; AUROC, area under ROC
